# Clinical characteristics and predictors of adverse outcomes in children with acute rheumatic fever from a high-endemic region of Brazil

**DOI:** 10.3389/fcvm.2026.1799209

**Published:** 2026-06-10

**Authors:** Renata Fonseca Mendoza, Letícia Leão de Oliveira, Bernardo Fonseca Mendoza, Jose Luiz Padilha da Silva, Paulo Henrique M. Melo, José Augusto Almeida Barbosa, Géssica Silva Santana, Airandes Pinto, Ndate Fall, Andrea Beaton, Maria Carmo Pereira Nunes

**Affiliations:** 1 Postgraduate Course of Infectious Diseases and Tropical Medicine, School of Medicine, Minas Gerais Federal University, Belo Horizonte, Minas Gerais, Brazil; 2 State Childreńs Hospital, Feira de Santana, Bahia, Brazil; 3 Department of Health, Feira de Santana State University, Feira de Santana, Bahia, Brazil; 4 Department of Statistics, Paraná Federal University, Curitiba, Paraná, Brazil; 5 Clinical Hospital, School of Medicine, Minas Gerais Federal University, Belo Horizonte, Minas Gerais, Brazil; 6 Cincinnati Children’s Hospital Medical Center, and the University of Cincinnati School of Medicine, Department of Pediatrics, Cincinnati, OH, United States

**Keywords:** acute carditis, acute rheumatic fever, erythrocyte sedimentation rate, mitral regurgitation, outcomes, predictors

## Abstract

**Introduction:**

Acute rheumatic fever (ARF) remains a leading cause of pediatric cardiovascular morbidity and a major driver of rheumatic heart disease (RHD) in low- and middle-income countries. Early identification of children at highest risk for adverse outcomes is essential to guide timely management and referral, but remains challenging in resource-limited settings. This study aimed to characterize the clinical profile of pediatric ARF and identify readily available predictors of adverse outcomes in a high-endemic region of Brazil.

**Methods:**

We conducted a cohort study of children treated for ARF at a tertiary pediatric center in Feira de Santana, Brazil, between 2010 and 2023, including both retrospectively identified cases and a prospectively enrolled subset through the Acute Rheumatic Fever Diagnosis Collaborative (ARC) Network. Clinical, laboratory, treatment, and outcome data were obtained from hospital and outpatient records. The primary outcome was a composite of cardiac surgery or death. Independent predictors were identified using multivariable logistic regression with covariate selection based on clinical relevance and model parsimony. Model performance was assessed using discrimination (C-statistic) and risk reclassification metrics.

**Results:**

A total of 151 children were included (median age, 9 years; 46% female). Carditis was present in 76%, with moderate-to-severe involvement in 49%, predominantly affecting the mitral valve. During a median follow-up of 17.5 months, 20 children (13%) experienced the primary outcome. In multivariable analysis, absence of fever (OR 0.14, 95% CI 0.31–0.65), higher ESR (OR 1.04 per mm/h, 95% CI 1.01–1.07), and lower hematocrit (OR 0.85, 95% CI 0.74–0.98) were independently associated with adverse outcomes. Incorporating erythrocyte sedimentation rate (ESR) into the model improved risk prediction, with a net reclassification improvement (NRI) of 0.79 (*P* = 0.005) and an integrated discrimination improvement (IDI) of 0.11 (*P* = 0.032). The final model demonstrated good discrimination (C-statistic 0.82). Results were consistent after multiple imputation.

**Conclusions:**

In children with ARF, simple inflammatory and hematologic markers are independently associated with progression to severe outcomes. ESR, an inexpensive and widely accessible test, provides incremental prognostic information and may support scalable risk stratification strategies aimed at reducing progression to advanced RHD in resource-limited endemic settings.

## Introduction

Acute rheumatic fever (ARF) is an autoimmune inflammatory disease triggered by group A *Streptococcus* infection that trigger rheumatic heart disease (RHD), a major contributor to cardiovascular morbidity and premature mortality worldwide (1–3). Beyond its long-term sequelae, ARF itself is associated with significant acute morbidity, including carditis, heart failure, arrhythmias, and, in severe cases, early death. Recurrent episodes further amplify cumulative cardiac injury, leading to progressive valvular damage, chronic disability, and increased risk of stroke and heart failure in later life (3, 4). Although largely preventable, RHD continues to disproportionately affect children and young adults in resource-limited settings, imposing a substantial global health and economic burden (4, 5).

The global prevalence of RHD has increased over the past decade, driven in part by expanded use of echocardiography and improved case detection (6, 7). Despite these advances, outcomes remain poor, as many patients are diagnosed at advanced stages, when secondary antibiotic prophylaxis has limited impact on disease progression (8–10). Accurate diagnosis during the acute phase remains challenging, as ARF often presents with nonspecific or subclinical features that overlap with other febrile illnesses, leading to missed opportunities for early initiation of secondary prophylaxis, when it has the greatest potential to prevent recurrent episodes and halt disease progression (8, 11, 12).

Adverse outcomes following ARF are strongly determined by the severity of initial cardiac manifestations, with moderate to severe carditis representing the most powerful predictor of long term morbidity and mortality (9, 13, 14). This underscores the critical need for practical tools capable of identifying mild disease at an early stage, before the development of severe cardiac involvement, particularly in high burden, resource-constrained settings.

Accordingly, contemporary characterization of ARF in endemic populations is essential to refine risk stratification and inform clinical decision-making. This study aimed to describe the clinical characteristics at diagnosis and to identify predictors of adverse outcomes in a cohort of children with ARF from a high-endemic region of Brazil.

## Methods

### Study population

This cohort study was conducted at the State Children's Hospital in Feira de Santana, Bahia, Brazil, a tertiary pediatric referral center serving 33 surrounding municipalities in the central-northern region of the state. Children diagnosed and treated for ARF between August 2010 and October 2023 were identified through hospital admission records. Beginning in July 2023, a subset of patients was prospectively enrolled through the Acute Rheumatic Fever Diagnosis Collaborative (ARC) Network, an international initiative that brings together global experts to discover and validate biomarkers for ARF and to support standardized patient identification and follow-up.

The diagnosis of ARF was established by the attending medical team in accordance with the Jones criteria and was subsequently reviewed by the principal investigator to ensure diagnostic consistency (15). Evidence of preceding group A *Streptococcus* infection was determined based on clinical history and/or elevated anti–streptolysin O (ASO) titers. Both initial and recurrent episodes of ARF were included in the analysis.

### Electrocardiographic and echocardiographic assessment

Electrocardiographic (ECG) data, when available, were extracted from medical records. All ECGs had been previously interpreted by pediatric cardiologists, with PR interval measurements documented. Atrioventricular conduction abnormalities were classified according to the attending cardiologist's assessment.

Transthoracic echocardiography was performed using commercially available ultrasound systems, and data were obtained from formal reports issued by pediatric echocardiographers. From 2012 onward, echocardiographic findings were interpreted in accordance with the 2012 World Heart Federation criteria for RHD (16). Extracted echocardiographic variables included the presence and severity of valvular regurgitation and stenosis, pericardial effusion, pulmonary hypertension, and left ventricular systolic function (17).

The severity of acute carditis was categorized as mild or moderate-to-severe based on the extent of valvular involvement, ventricular function, and associated hemodynamic consequences. Moderate-to-severe carditis was defined by the presence of moderate or severe mitral regurgitation, mitral stenosis, or aortic regurgitation, with or without concomitant heart failure.

### Adverse outcomes

The primary adverse outcome was a composite of cardiovascular death or cardiac surgery occurring during the acute phase or within one year of ARF diagnosis. Surgical interventions included valve repair or replacement and were indicated based on severe valvular dysfunction with hemodynamic compromise, refractory heart failure, pulmonary hypertension, or left ventricular dysfunction. Decisions regarding surgery were made by the treating multidisciplinary team.

Follow-up data were obtained from medical records and supplemented by direct contact with family members when necessary. The study was approved by the institutional ethics committee, adhering to ethical standards for human research (Protocol No. 6.639.220).

### Statistical analysis

Continuous variables are presented as mean ± standard deviation, and categorical variables as frequencies and percentages. Categorical comparisons were performed using the chi-square test. Multivariable logistic regression was used to identify factors independently associated with the composite outcome. Clinically relevant variables were selected *a priori*. Model calibration was assessed using the Hosmer–Lemeshow test, and discrimination using the C-statistic.

The incremental prognostic value of erythrocyte sedimentation rate (ESR) was evaluated using net reclassification improvement (NRI) and integrated discrimination improvement (IDI). Continuous NRI was applied to avoid arbitrary risk categorization and capture changes in predicted risk for both events and non-events.

Missing data were present for C-reactive protein (*n* = 12), erythrocyte sedimentation rate (*n* = 26), hematocrit (*n* = 7), and ARF recurrence (*n* = 24). Multiple imputation by chained equations was performed under the assumption of missing at random. Continuous variables were imputed using linear regression models, and binary variables using logistic regression models. The imputation model included age, sex, fever, ARF recurrence, C-reactive protein, erythrocyte sedimentation rate, hemoglobin, and hematocrit. A large number of imputations (m = 1,000) was performed to ensure stability of estimates, and estimates were combined using Rubin's rules. Model discrimination across imputed datasets was summarized using the median C-statistic and corresponding empirical 95% confidence intervals (2.5th–97.5th percentiles).

Internal validation was performed using bootstrap resampling with 1,000 samples to estimate optimism-corrected performance, including discrimination and calibration.

Statistical analyses were performed using SPSS version 22.0 (IBM Corp., Chicago, IL, USA) and R version 4.5.3 (R Foundation for Statistical Computing, Vienna, Austria), using the packages *tidyverse*, *ggalluvial*, *rms*, *pROC*, *PredictABEL*, and *mice*.

## Results

### Baseline characteristics

**Figure 1:**
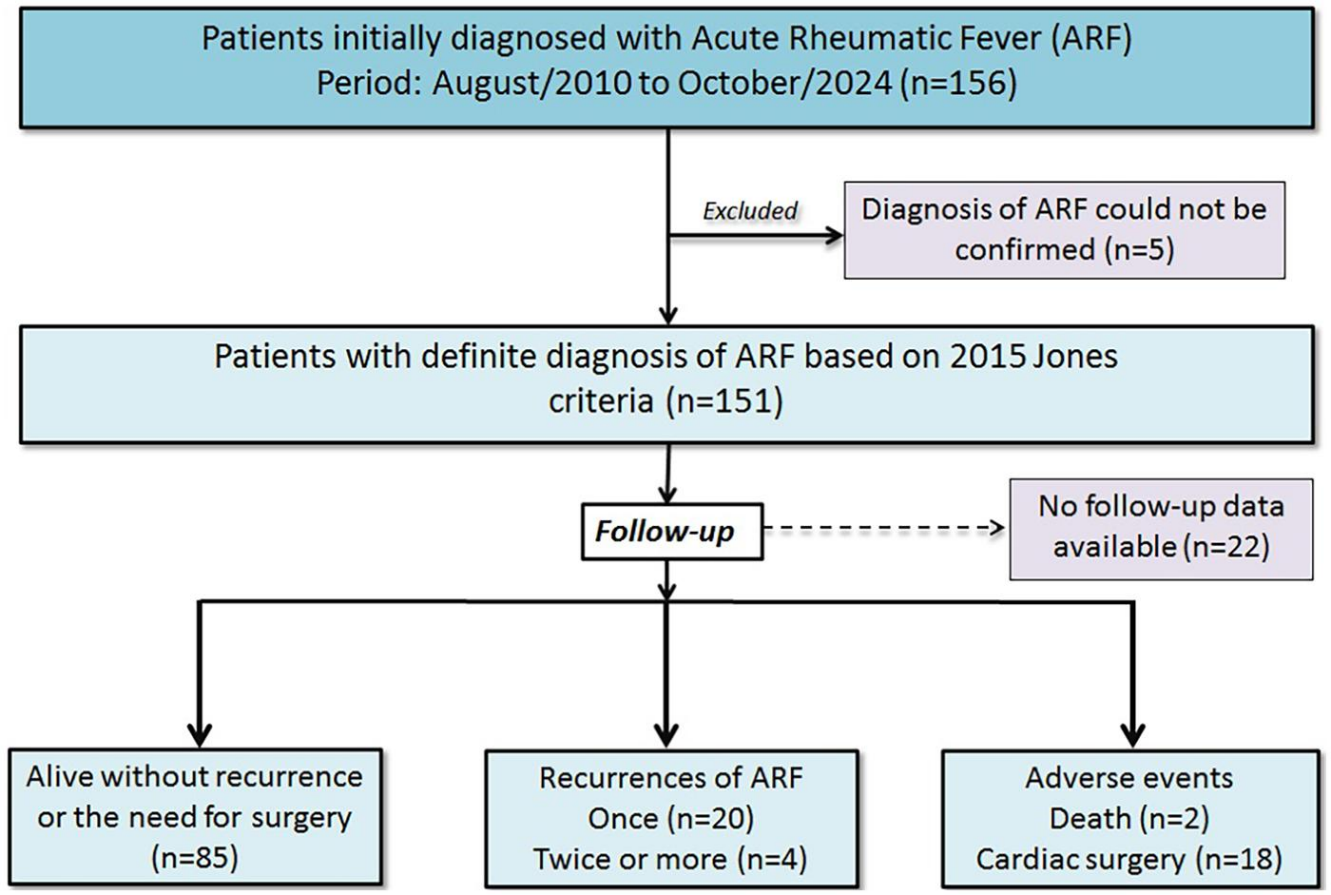
Flow diagram of patient selection and study population.

The cohort included 151 children (Figure 1), of whom 121 (80%) were recruited retrospectively and 30 (20%) prospectively. The median age at diagnosis was 9 years (IQR 7–12; range 2–15 years), and 69 (46%) were female. Baseline characteristics are summarized in Table 1. At enrollment, 137 patients (91%) presented with their first episode of ARF, while 14 (9%) had a prior diagnosis of the disease and were included during a recurrence. A family history of ARF was reported in 13 patients (9%). Comorbid conditions were uncommon and included sickle cell disease (*n* = 4, 3%), prior atrial septal defect repair (*n* = 1), and isolated cases (≈1% each) of obesity, type 1 diabetes, immune thrombocytopenia, epilepsy, and neurofibromatosis.

**Table 1:** Baseline clinical characteristics of the study population.

Variables	Number (%) or median (IQR)
Age (years)	9 (7–12)
Female	69 (46%)
Positive family history	13 (9%)
Jones criteria	
Streptococcal evidence	108 (72%)
Positive antistreptolysin O	89 (59%)
Major Jones criteria	
Carditis	
Mild	59 (51%)
Moderate/Severe	56 (49%)
Arthralgia/Arthritis	78 (52%)
Monoarthritis	8 (10%)
Polyarthritis	49 (63%)
Polyarthralgia	21 (27%)
Chorea	49 (33%)
Subcutaneous nodules	6 (4%)
Erythema marginatum	9 (6%)
Number of major Jones criteria met	
One	68 (45%)
Two	68 (45%)
Three	15 (10%)
Minor Jones criteria	
Fever	85 (56%)
Monoarthralgia	6 (4%)
ESR ≥ 30 mm/h	39 (26%)
ESR value (mm/h)	22 (13.5–33.5)
C-reactive protein > 5 mg/L	96 (64%)
C-reactive protein value (mg/L)	16.1 (5–64.4)
PR prolongation	7 (5%)
Number of minor Jones criteria met	
None	33 (22%)
One	42 (28%)
Two	67 (44%)
Three	9 (6%)

**Figure 2:**
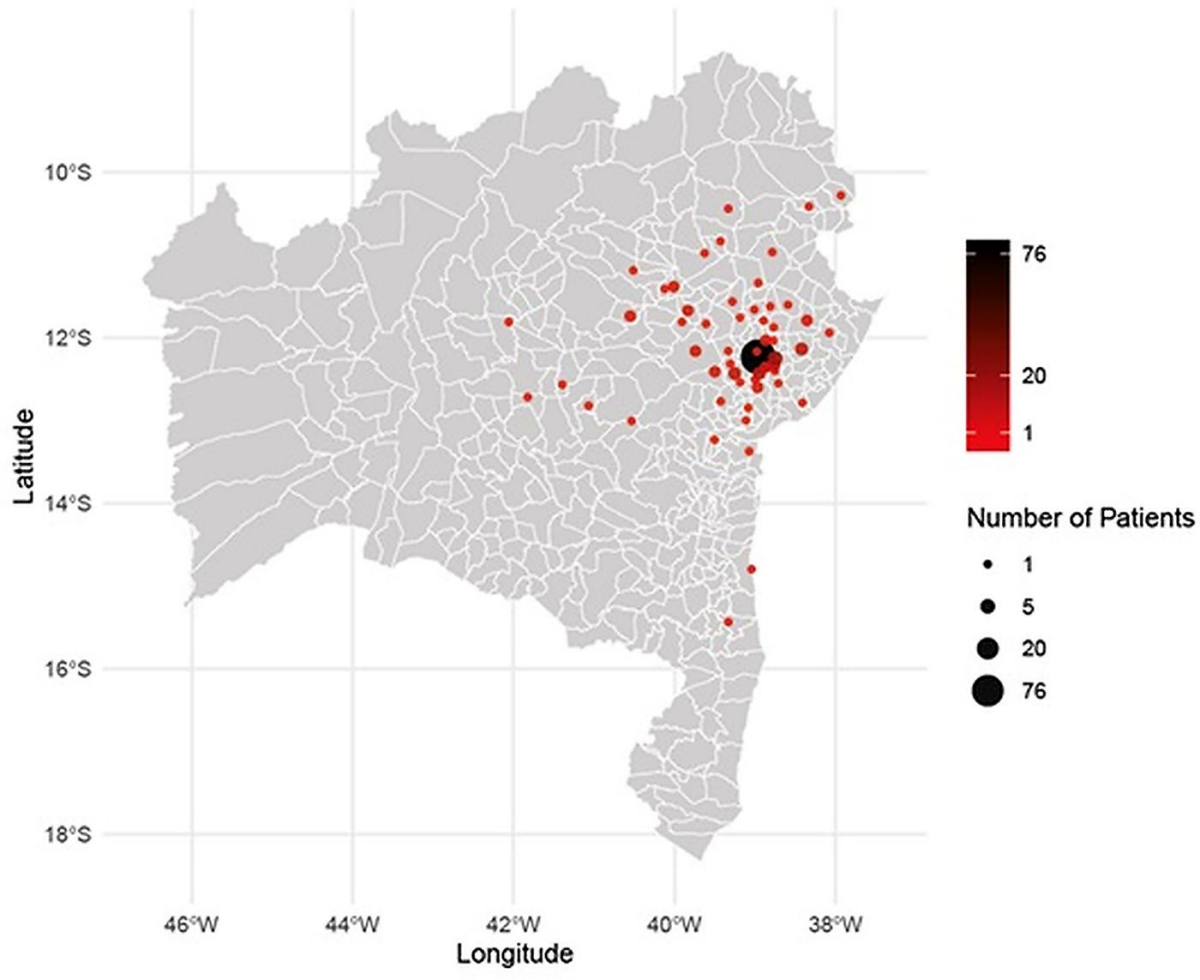
Geographic distribution of patients according to city of origin within the state of Bahia, Brazil, showing concentration near the eastern area. Dot size and color correspond to patient count.

Patients originated from 55 municipalities, with approximately half residing in the referral city (Figure 2). Evidence of preceding group A *Streptococcus* infection was documented in 108 patients (72%), based on clinical history and/or elevated anti–streptolysin O (ASO) titers. Elevated ASO levels were present in 89 children (59%), with a median titer of 400 IU/mL (IQR 199–600).

Carditis was the most frequent major Jones criterion, occurring in 115 patients (76%), with moderate-to-severe involvement in 56 (49%). The mitral valve was most commonly affected (76%), with mild, moderate, and severe regurgitation observed in 37%, 10%, and 29%, respectively. Mitral stenosis at presentation was infrequent (5%) and uniformly mild. Aortic valve involvement occurred in 48 patients (33%), including severe regurgitation in 9 (6%). Pulmonary hypertension was present in 19 patients (13%), and pericardial effusion in 10 (7%), none requiring intervention.

Joint involvement was observed in 78 patients (52%), most commonly as polyarthritis. Sydenham chorea occurred in 49 patients (33%). Erythema marginatum and subcutaneous nodules were uncommon (6% and 4%, respectively). Fever was reported in 85 patients (56%). Inflammatory markers were elevated in 105 patients (70%), including elevated C-reactive protein in 64% and elevated erythrocyte sedimentation rate (ESR) in 26%. PR interval prolongation was documented in 7 patients (5%).

### Treatment and clinical course

Hospitalization was required in 132 patients (87%), including 19 (14%) admitted to the intensive care unit. Median length of stay was 12 days (IQR 7–27). During the acute phase, 5 patients underwent cardiac surgery, and 13 additional patients were referred for surgery due to severe valvular disease (Table 2).

**Table 2:** Clinical outcomes of the study population.

Variables	Number (%) or median (IQR)
Hospital admission	132 (87%)
Length of stay	12 (7–27)
Intensive care unit	19 (14%)
Follow-up time (months)	17.5 (5–51.5)
Heart failure	8 (6%)
Recurrence	
No	103 (81%)
Once	20 (16%)
Twice or more	4 (3%)
Complications	
Pulmonary hypertension	8 (6%)
Endocarditis	2 (2%)
Endocarditis and embolic event	3 (2%)
Cardiac surgery	
Mitral valve repair	15 (83%)
Aortic valve repair	1 (6%)
Mecanic aortic valve replacement	2 (11%)
Tricuspid repair	1 (6%)
Unknown	3 (17%)
Death	2 (1%)

Secondary prophylaxis with benzathine penicillin G was initiated in 136 patients (90%), and 66 (44%) received corticosteroids. Adjunctive therapies included diuretics and angiotensin-converting enzyme inhibitors for heart failure, haloperidol for chorea, and aspirin or nonsteroidal anti-inflammatory drugs for arthritis.

**Figure 3:**
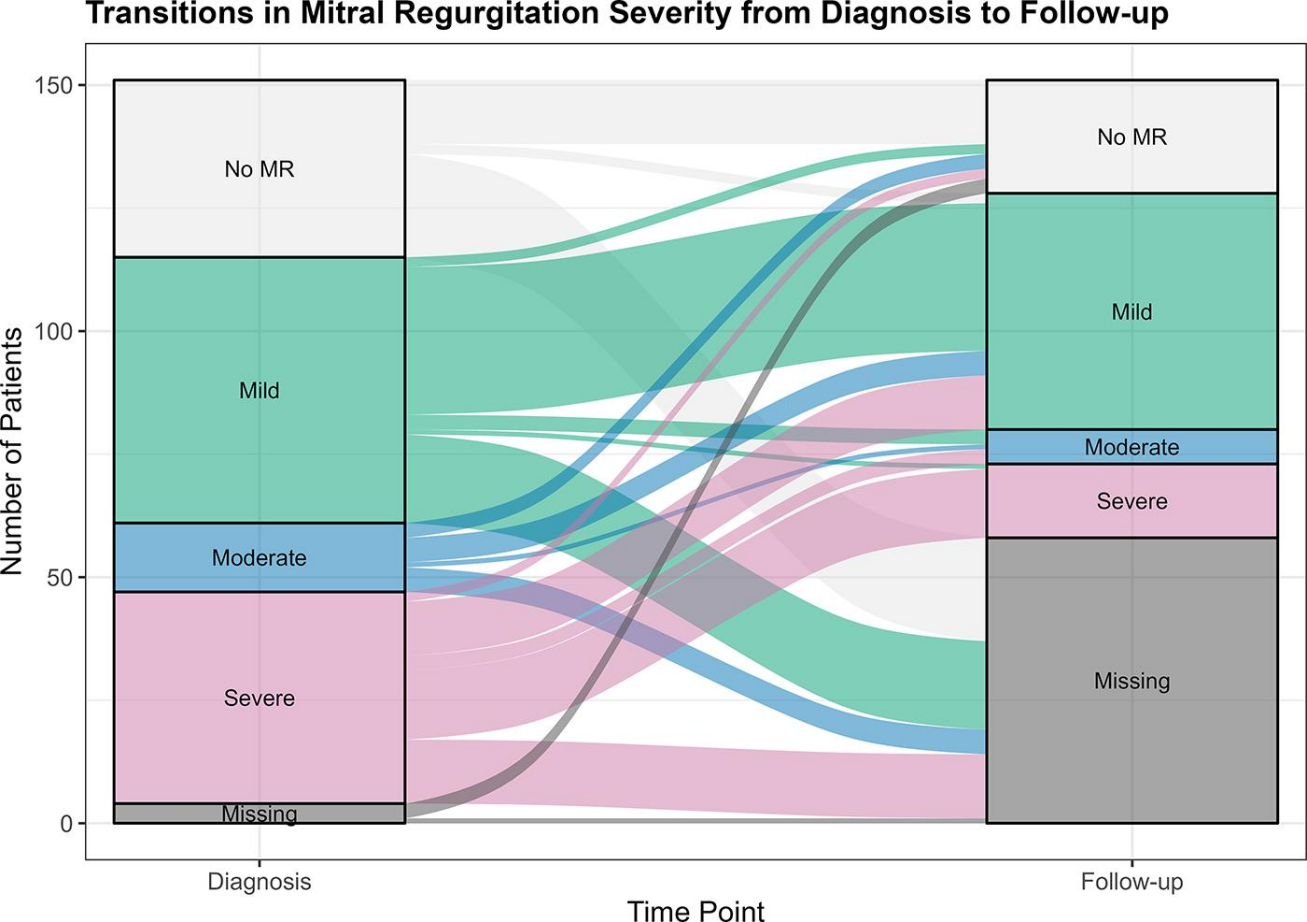
Transitions in mitral regurgitation severity from diagnosis to follow-up. Sankey plot illustrating changes in mitral regurgitation (MR) severity between baseline (diagnosis) and follow-up. Categories include no MR, mild, moderate, severe, and missing data. The width of each stream represents the number of patients transitioning between severity categories over time.

Follow-up echocardiography was available in 93 patients. Mitral regurgitation remained the most prevalent lesion, observed in 70 patients, with severe regurgitation persisting in 15 (29%). Aortic valve involvement was present in 30 patients, and mitral stenosis developed or progressed in 6 patients (Figure 3). Most patients with mild mitral regurgitation at baseline remained stable, whereas severe regurgitation frequently persisted despite therapy.

### Predictors of adverse outcome

A total of 24 patients were lost to follow-up; outcome data were available for 127 patients (84%), with a median follow-up of 17.5 months (IQR: 5.0–51.5). ARF recurrence occurred in 24 patients (19%), including 4 with multiple recurrences (Figure 1). During a median follow-up period, 18 patients (12%) required cardiac surgery, most commonly due to chordae tendineae rupture with severe mitral regurgitation. Two patients died during hospitalization from cardiogenic shock. The incidence rate of adverse events was 6.48 per 100 person-years (95% CI: 3.96–9.96).

At last follow-up, 8 patients (6%) had persistent heart failure symptoms, 8 had pulmonary hypertension, and 5 developed infective endocarditis, including 3 with embolic complications.

Inclusion of ESR significantly improved risk prediction, with a continuous net reclassification improvement of 0.79 (95% CI 0.24–1.34; *p* = 0.005) and an integrated discrimination improvement of 0.11 (95% CI 0.009–0.20; *p* = 0.033).

In multivariable analysis, absence of fever at presentation, higher ESR, and lower hematocrit were independently associated with adverse outcomes (Table 3). The final model demonstrated good discrimination (C-statistic 0.82; 95% CI 0.69–0.96) and adequate calibration (Hosmer–Lemeshow *χ*^2^ = 7.34, *p* = 0.50). In internal validation, optimism-corrected discrimination remained similar (C-statistic 0.80; 95% CI 0.68–0.93), although the calibration slope (0.86) suggested some degree of overfitting, likely related to the limited number of events. Analyses based on multiple imputation yielded consistent results.

**Table 3:** Predictors of adverse outcomes in children with acute rheumatic fever.

At baseline	Univariable analysis		Multivariable analysis*		Multiple imputation analysis**	
	Odds ratio (95% CI)	Value	Odds ratio (95% CI)	P value	Odds ratio (95% CI)	P value
Age, years	1.048 (0.902–1.219)	0.540	…	…	…	…
Female sex	1.947 (0.746–5.081)	0.173	…	…	…	…
Fever	0.282 (0.102–0.781)	0.015	0.142 (0.031–0.654)	0.012	0.177 (0.047–0.673)	0.011
ARF recurrence	1.074 (0.385–2.997)	0.892	…	…	…	…
Antistreptolysin O	0.999 (0.997–1.002)	0.603	…	…	…	…
C-Reactive Protein	0.996 (0.985–1.007)	0.483	0.987 (0.973–1.002)	0.090	0.987 (0.973–1.002)	0.091
Erythrocyte Sedimentation Rate	1.023 (1.002–1.044)	0.032	1.038 (1.011–1.066)	0.006	1.035 (1.008–1.063)	0.011
Hemoglobin (Hb)	0.762 (0.581–0.999)	0.049	…	…	…	…
Hematocrit (Ht)	0.908 (0.830–0.994)	0.036	0.849 (0.738–0.976)	0.021	0.848 (0.748–0.961)	0.010
Pericardial effusion	4.055 (0.934–17.606)	0.062	…	…	…	…

* Complete case analysis. Multivariable model C-statistic: 0.822 (95% CI 0.693–0.952).

** Multiple imputation analysis. Multivariable C-statistic: 0.814 (95% CI 0.762–0.851).

## Discussion

In this contemporary pediatric cohort of ARF, adverse outcomes occurred in more than one in ten patients within a relatively short follow-up, confirming the persistent severity of this disease despite modern diagnostic and therapeutic strategies. Carditis, particularly moderate-to-severe mitral valve involvement, dominated the clinical presentation and remained the principal driver of surgical intervention and mortality. Beyond structural disease severity, we identified a distinct clinical and inflammatory profile at presentation associated with poor outcomes, characterized by absence of fever, elevated ESR, and lower hematocrit. Notably, incorporation of ESR significantly improved risk discrimination and reclassification, highlighting its incremental prognostic value over traditional clinical markers. Together, these findings emphasize that readily available bedside and laboratory parameters can refine early risk stratification in pediatric acute rheumatic fever and may help identify children at highest risk for progression to severe RHD.

### Clinical presentation of ARF

The median age at diagnosis in this cohort was 9 years, consistent with contemporary pediatric ARF series across diverse endemic and non-endemic regions, including Latin America, sub-Saharan Africa, and the Middle East (5, 9, 11). The near-equal sex distribution aligns with established epidemiology, supporting the concept that ARF affects boys and girls similarly (9, 11, 18, 19). Notably, although Sydenham chorea has been reported to predominate in females (20), our cohort did not demonstrate a female excess, suggesting that sex-related differences in neurological involvement may vary across populations or be influenced by case ascertainment and referral patterns.

Carditis was the dominant clinical manifestation, affecting more than three-quarters of patients, with nearly half presenting with moderate-to-severe disease. This high burden of cardiac involvement underscores the severity of ARF at presentation in contemporary endemic settings and likely reflects delayed recognition, recurrent episodes, or limited access to early care (5, 18, 21).

Although some improvement in mitral and aortic regurgitation was observed on follow-up echocardiography, consistent with prior reports of partial recovery after effective secondary prophylaxis (8), severe and persistent valvular disease remained common, reinforcing the long-term impact of acute inflammatory injury (13). However, the higher loss to follow-up among patients with milder valvular lesions may have led to an overestimation of disease persistence in our cohort.

Arthritis was the second most frequent major manifestation but occurred less often than reported in several prior cohorts (22, 23). Importantly, monoarticular presentations accounted for a meaningful proportion of cases, emphasizing that atypical joint involvement remains clinically relevant and may contribute to underdiagnosis when classical migratory polyarthritis is absent (24).

### Mechanistic insights into the “afebrile but inflamed” phenotype

One of the most striking findings of this study was the inverse association between fever at presentation and adverse outcomes, particularly when coupled with elevated inflammatory markers and anemia. This “afebrile but inflamed” phenotype likely reflects a distinct pathophysiological trajectory in ARF. Afebrile presentations may reflect a more insidious clinical course, potentially leading to delayed healthcare seeking and diagnosis, and consequently more advanced cardiac involvement at presentation.

Fever is often associated with acute, systemic inflammatory responses and prominent extracardiac manifestations, particularly arthritis, which often facilitate earlier clinical recognition and treatment (7, 15, 25). In contrast, the absence of fever may identify a subgroup with a less overt but more cardiac-predominant disease phenotype, characterized by attenuated systemic symptoms and a higher likelihood of delayed diagnosis. In this setting, inflammatory activity may already be established at the myocardial and valvular level, despite minimal systemic manifestations.

Moreover, the lack of fever at presentation does not preclude a preceding inflammatory trigger. Some patients may have experienced an earlier episode of streptococcal pharyngitis or febrile illness that was unrecognized or inadequately treated, with cardiac involvement emerging as the dominant clinical manifestation weeks later. This temporal dissociation may contribute to the under-recognition of early disease stages, during which subclinical carditis could already be detectable by echocardiography.

Elevated ESR in these patients likely reflects sustained immune activation, driven by high fibrinogen levels, immune complex deposition, and ongoing endothelial and valvular inflammation (17, 26). Unlike C-reactive protein, which responds rapidly to acute cytokine signaling, ESR may better capture cumulative or prolonged inflammatory burden, particularly in subacute or smoldering disease (24). This may explain why ESR, but not CRP, demonstrated stronger and independent prognostic value in this cohort (26, 27).

Our findings differ from those of Licciardi et al. (28), who reported an association between higher ESR and regression of mitral regurgitation in a low-severity Italian cohort. This discrepancy likely reflects differences in disease spectrum, as their population had low hospitalization rates and no reported surgical interventions or deaths, whereas our cohort included a high proportion of hospitalized patients with severe cardiac involvement.

The concomitant presence of low hematocrit further supports this high-risk inflammatory phenotype. Anemia in ARF is multifactorial, resulting from inflammation-mediated suppression of erythropoiesis, altered iron metabolism, and increased metabolic demand. In the setting of carditis, reduced oxygen-carrying capacity may exacerbate myocardial stress and amplify the hemodynamic consequences of valvular regurgitation. Taken together, absence of fever, elevated ESR, and low hematocrit may identify children with advanced inflammatory cardiac involvement who lack early clinical warning signs yet are at highest risk for disease progression.

### Determinants of poor prognosis in ARF

Consistent with prior literature, the severity of carditis emerged as the central determinant of adverse outcomes, including heart failure, need for surgical intervention, and mortality (17, 29). Recurrent ARF episodes further compounded risk, reinforcing the critical role of sustained secondary prophylaxis (13, 21).

Beyond structural severity, our findings extend existing knowledge by demonstrating that readily available laboratory markers, ESR and hematocrit, provide incremental prognostic information. Notably, incorporation of ESR significantly improved risk discrimination and reclassification, highlighting its potential utility as a low-cost tool for early risk stratification. These markers are particularly attractive in resource-limited settings, where access to advanced imaging or biomarkers may be constrained.

Despite a substantial burden of severe disease, surgical outcomes in our cohort were favorable, with good postoperative recovery. Mortality was low compared with reports from other endemic regions, likely reflecting access to specialized cardiac care (21). Nevertheless, the need for surgery in childhood underscores the long-term consequences of delayed diagnosis and inadequate prevention (8, 10).

Although ARF and RHD are largely preventable, a substantial proportion of cases remain unrecognized during the acute phase, limiting opportunities for secondary prophylaxis. Strengthening early diagnostic pathways, improving clinical awareness of atypical presentations, and ensuring sustained access to prophylactic programs remain central to reducing the global burden of RHD (4, 17).

### Study limitations

Several limitations merit consideration. First, the retrospective component of the cohort introduces potential selection and information biases, including reliance on medical records and diagnostic coding. Second, echocardiographic assessment of carditis severity was based primarily on valvular regurgitation severity, without systematic characterization of valve morphology or mechanisms of regurgitation, which may further refine prognostic stratification. Third, follow-up echocardiography was not uniformly available for all patients, particularly those with mild disease, potentially limiting assessment of long-term valvular progression. Finally, although multivariable modeling was performed, residual confounding cannot be excluded.

## Conclusions

In this contemporary pediatric cohort with ARF, absence of fever, elevated ESR, and low hematocrit at presentation identified children at increased risk for adverse outcomes. ESR provided incremental prognostic value beyond traditional clinical markers, supporting its role as an accessible and cost-effective tool for early risk stratification. These findings highlight the importance of recognizing atypical, afebrile inflammatory presentations of ARF and reinforce the need for timely diagnosis, sustained prophylaxis, and equitable access to specialized care to reduce progression to severe RHD.

## Data Availability

The raw data supporting the conclusions of this article will be made available by the authors, without undue reservation.
